# Analysis and Comparison of Natural Shear and Induced Tensile Fractures for Caprock Leakage Assessment

**DOI:** 10.1007/s11242-025-02283-0

**Published:** 2026-01-14

**Authors:** Sahyuo Achuo Dze, Tomos Phillips, Reza Najafi-Silab, Sarah Perez, Tom Bultreys, Vladimir Novak, Christian M. Schlepütz, Veerle Cnudde, Florian Doster, Kamaljit Singh, Kevin Bisdom, Andreas Busch

**Affiliations:** 1 Lyell Centre, Heriot-Watt University, Edinburgh, UK; 2 Institute of GeoEnergy Engineering, Heriot-Watt University, Edinburgh, UK; 3 Centre for X-Ray Tomography (UGCT), Ghent University, Ghent, Belgium; 4 Swiss Light Source, Paul Scherrer Institute, Villigen, Switzerland; 5 Department of Earth Sciences, Utrecht University, Utrecht, The Netherlands; 6 Shell Global Solutions International BV, The Hague, The Netherlands

**Keywords:** Fracture flow, Caprock, Shear, Tensile, Heterogeneity, Simulation

## Abstract

The comparison between laboratory-induced and subsurface fractures, and their corresponding flow is still unclear. Here, we examine three natural shear fractures and two induced tensile fractures from the same low-permeability lithology. Using high-resolution synchrotron imaging, we extracted three-dimensional fracture void geometries to analyse aperture distributions, surface roughness, and spatial correlation patterns. We then compared measured fracture transmissivities against theoretical predictions from parallel-plate models (cubic law) and direct numerical simulations (DNS) of flow to evaluate consistency and uncertainties. We find that despite differences in heterogeneity, induced tensile and natural shear fractures can generally yield similar flow in caprocks. Our comparisons further indicate that the choice of flow estimation method can introduce more uncertainty than the fracture opening mode.

**Supplementary Information:**

The online version contains supplementary material available at 10.1007/s11242-025-02283-0.

## Article Highlights

- For fractures occurring in the same caprock lithology, permeability is similar for shear and induced fractures
- Laboratory-induced fractures can serve as analogues for natural ones in caprock leakage assessment.

**Supplementary Information:**

The online version contains supplementary material available at 10.1007/s11242-025-02283-0.

## Introduction

### Background

Fractures can serve as primary conduits for subsurface fluid migration, significantly influencing permeability, storage capacity, and leakage potential in low-permeability formations such as caprocks. In clay-rich caprocks, matrix permeability is often negligible, making fracture networks the dominant pathways for fluid transport (Neuzil 2019). These are critical for subsurface systems including hydrocarbon production, hydrogen and carbon storage sites where coupled thermo–hydro–mechanical (THM) processes alter stress, pore pressure and temperature in the reservoir–caprock system. THM and hydro-geomechanical simulations have shown that injection-induced pressure and thermal changes can open, close or reactivate fractures and faults, modifying caprock sealing capacity and potentially creating leakage pathways or inducing seismicity (Vilarrasa 2016; Vilarrasa and Rutqvist 2017). In all such settings, the way fluids move through fractures in the caprock is controlled by the geometry and connectivity of those fractures at the scale of individual apertures. Therefore, understanding fluid transport behaviour within caprock fractures is essential for predicting long-term containment integrity and leakage.

Fractures are broadly classified based on the mode of rock failure that produced them (tensile or shear) and origin (natural or induced). Natural shear fractures, typically associated with faulting, develop where differential stress results in displacement along a fracture plane. They are common in tectonic deformation zones and can significantly impact permeability if they remain open or are reactivated under current stress conditions (Zhang et al. 2018). Tensile fractures, on the other hand, occur when rock is pulled apart due to stresses that exceed the rock’s tensile strength. These can arise naturally from processes such as overpressure, disequilibrium compaction, or hydrocarbon generation, and can also be induced by operational activities such as hydraulic fracturing or drilling (Cosgrove 2001; Zoback 2007). Although both natural shear and tensile fractures can occur in the same caprock, their spatial distributions often vary. In nature, shear fractures are frequently concentrated in fault zones, whereas tensile fractures can be prevalent away from major faults, where local extensional regimes or fluid overpressures dominate.

Prior research (Busch and Kampman 2018; Rizzo et al. 2024; Snippe et al. 2022) has extensively examined caprock seal integrity and leakage risk. However, detailed geometric characterization of individual fractures in weak, fine-grained caprocks remains limited. Clay-rich mudstones and claystones present sampling and handling challenges, as they easily disintegrate (Ewy 2015; Phillips et al. 2020). As a result, much of the classical fracture characterization work has focussed on harder, more competent rocks like granites, sandstones, or carbonates (Table 1). Moreover, non-destructive high-resolution imaging (e.g. micro-CT) providing the opportunity to directly measure void geometry and relate it to flow, has only recently been applied to caprock fractures.

**Table 1:** Selected single fracture studies, highlighting the methods and comparative analysis that are relevant to the current study

Author(s) & Year	Rock type	Fracture type	Aperture measurement method	Study objectives
This Study	Claystone	Drilling-induced tensile & Natural shear	Digital extraction of fracture void and pointwise (every 2.75 µm) measurement via Euclidean distance transform	Quantitative characterisation and comparison of fracture types in a claystone caprock
Cunha et al. (2024)	Gneiss	Lab-induced tensile	Surface profilometry and ‘re-matching’ to obtain apertures for every 1.25 mm	Upscaled aperture fields using spatial continuity
Marliot et al. (2024)	Cement mortar	Lab-induced shear	Optical & scanning electron microscopy (SEM), XCT, digital volume correlation	Quantitative aperture characterization and comparative imaging
Kurotori et al. (2023)	Basalt	Natural tensile	PET inversion, missing-attenuation, X-CT calibration	Comparative analysis of methods
Vandersteen et al. (2003)	Limestone	Natural tensile	X-CT calibration, identification of ‘missing rock mass’ in pixels, thus apertures	3D aperture distribution and flow analysis in limestone
Ogilvie et al. (2003)	Granite, Syenite, Gabbro	Lab-induced tensile	Optical profiling of individual fracture surfaces, combined to obtain apertures for every 15–30 µm	Characterizing rough-walled fractures in crystalline rock
Keller (1998)	Granite, Sandstone	Natural tensile	X-CT calibration, identification of ‘missing rock mass’ in pixels, thus apertures (min. 35 µm)	High‑resolution aperture measurement and morphological characterization
Hakami and Larsson (1996)	Granite	Natural tensile	Manual profile cuts along core and microscope-based apertures obtained every 50–200 µm	Aperture characterization correlated with fluid flow predictions
Hakami et al. (1995)	Granodiorite	Natural tensile & Lab-induced tensile	Injection of fluid, casting, and surface topography	Comparison of methods for obtaining aperture distributions
Hakami (1995)	Granite	Natural shear (minor fault)	Resin injection whose thickness estimated apertures	Aperture distribution, heterogeneity, and connectivity
Iwano and Einstein (1993)	Granite, Andesite, Sandstone, Schist	Natural tensile & Lab-induced tensile	Optical profilometry, resolved at 5 µm of fracture surfaces, which are overlapped numerically to obtain apertures	Spatial correlation of fracture apertures across rock types
Cox and Wang (1993)	Granite	Natural tensile & shear	Ligh transmission through dyed fluid, whose thickness is equivalent to the aperture	Quantitative differences in aperture patterns
Pyrak-Nolte et al. (1987)	Granite	Natural shear	Metal injection and SEM to characterise apertures based on metal filling	Hydromechanical properties of natural fractures in low-permeability rock

A selection of relevant single-fracture studies in the literature, highlighting rock and fracture types, aperture measurement method, and objectives is shown in Table 1.

These studies have typically considered only one fracture type at a time or examined fractures in different rock types, making comparisons ambiguous. Gale (1982) initiated a comparison by investigating the stress-permeability relationships of natural and induced fractures, highlighting key factors that influence fluid flow in fractured rock. Other classic investigations (Cuss et al. 2015; Haller and Porturas 1998; Kulander et al. 1990; Lorenz and Cooper 2017) emphasized qualitative differences in morphology between natural and induced fractures (e.g. differences in roughness or wall matching) without quantifying how those differences impact fluid flow properties (e.g. permeability). As Table 1 indicates, only a handful of studies have attempted such quantitative comparisons, and none have done so for the same fine-grained lithology.

### Motivations

Predicting caprock leakage is complicated by uncertainties in how well laboratory or model fractures can predict actual subsurface fracture flow. Natural subsurface shear fractures in caprocks are often more complex than their experimental or modelled counterparts and are difficult to sample. In contrast, tensile fractures can easily be induced in the laboratory and readily used for flow studies. Comparing these two fracture types allows us to assess the influence of fracture origin and morphology on flow and evaluate the effectiveness of various modelling approaches. This brings us to two main hypotheses.

First, we hypothesize that geometric differences arising from fracture formation mode result in measurable differences in flow behaviour. Specifically, natural shear fractures are expected to exhibit more variable aperture distributions and poorer matedness (match between fracture walls) compared to induced tensile fractures, potentially leading to lower effective flow rates. However, the magnitude of this effect in caprocks remains uncertain, as the fine grains and clay-rich composition may limit fracture surface roughness, making induced and natural fractures more alike than in coarse-grained rocks.

Secondly, we hypothesize that the choice of estimating flow (e.g. applying a parallel-plate cubic law, performing direct numerical simulations, or conducting laboratory measurements) can introduce significant variability in permeability predictions, potentially exceeding the differences attributable to fracture type alone. In other words, a smooth tensile fracture and a rough shear fracture might carry similar flow under certain conditions, but oversimplified modelling approaches could substantially overestimate flow rates for both.

By combining high-resolution 3D imaging with flow experiments and numerical simulations on both natural and induced fractures from the same caprock lithology, we analyse fracture geometry and compare permeability estimation methods. Our goal is to determine how well an induced tensile fracture can serve as a proxy for natural shear fractures in terms of flow performance, while quantifying the effectiveness of different modelling approaches.

To our knowledge, no previous study has integrated high-resolution imaging data, flow measurements, and simulations to directly compare shear and tensile fractures in a clay-rich caprock. Ultimately, this work aims to reduce uncertainty and improve predictive models of fracture-controlled flow in caprocks, enhancing our ability to evaluate leakage risk and ensure long-term containment in engineered geological environments. The modes of failure and effects of fracture flow on failure are not addressed.

## Materials and Methods

### Sample Origin

**Fig. 1:**
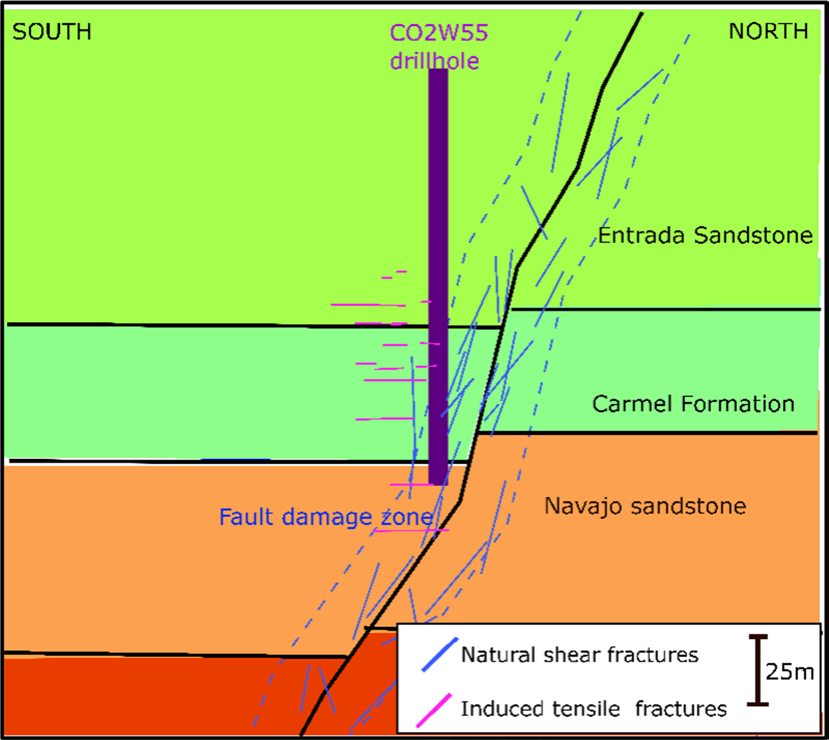
Schematic of the Little Grand Wash Fault System near Green River, Utah, showing the drillhole into the damage zone from which core samples were obtained for this study (adapted from Kampman et al. (2014)). Induced tensile fractures may interact with pre-existing natural shear fractures within the formations

The rock samples analysed in this study originate from the Jurassic Carmel Formation, a ~ 50 m thick shallow-marine sequence composed of interbedded red and grey claystone, gypsum, siltstone, and fine-grained sandstone. Core samples were obtained from the CO2W55 well, which intersects the Carmel caprock and the Little Grand Wash Fault damage zone near Green River Basin, Utah (Kampman et al. 2014) (Fig. 1).

### Overview of Experiment

For flow experiments and imaging, five cylindrical core sample plugs (18 mm in length, 6 mm in diameter) were extracted from larger rock specimens. These sample dimensions were dictated by the requirements of the flow apparatus (Phillips 2022; Phillips et al. 2024). The selected subsamples include:

(i) Three natural shear fractures (NFs) interpreted to have formed in situ during faulting. These natural fractures exhibit visible shear displacement and rough, mismatched fracture surfaces.
(ii) Two induced tensile fractures (IFs)—most likely induced during drilling or core handling. They are tensile splits through the rock, generally lacking shear offset.

After extraction, each mini-core sample contained a single dominant fracture roughly going through the cylindrical core. The fractures were interpreted to be open, with no report of filling or mineralization. In preparation for imaging, samples were mounted in a small pressure vessel. To mimic in situ stress conditions and preserve fracture aperture characteristics, imaging was conducted under an isotropic confining stress of 45 bar and a pore pressure of 20 bar, yielding an effective stress of approximately 25 bar across the fracture during scanning. Maintaining this effective stress ensured that fracture apertures in the lab remained representative of field conditions (avoiding unrealistically wide openings due to core relaxation), although temperature was maintained at room temperature (~ 25 °C) It should be noted that while the synchrotron imaging apparatus was part of multi-phase flow experiments, here we focus exclusively on the fracture geometries under water-saturated, single-phase conditions. Some fracture metrics such as aperture are stress-dependent; thus, the distributions observed here may evolve under different stress conditions. Investigating how stress changes might differentially affect natural vs. induced fractures (e.g. their closure behaviour) is beyond our current scope but is identified as an important avenue for future work.

High-resolution synchrotron X-ray computed tomography (XCT) was performed at the TOMCAT beamline (X02DA), Swiss Light Source, Paul Scherrer Institute, Switzerland. Each 3D tomographic scan consisted of 2000 angular projection images over 180°, with a voxel size of 2.75 µm. The fine resolution enabled us to clearly resolve the space between fracture walls (void) and the surface details. This level of detail implies that the field of view, thus images, is limited to the middle part of the 18-mm-long core (~ 5 mm). The reconstructed µCT image volumes capture the full fracture geometry in three dimensions, providing the basis for detailed quantitative analysis of fracture aperture, surface roughness, and spatial variability. Full details of the experimental and setup procedure are provided in Phillips (2022) and Phillips et al. (2024).

### Data and Image Analysis

**Fig. 2:**
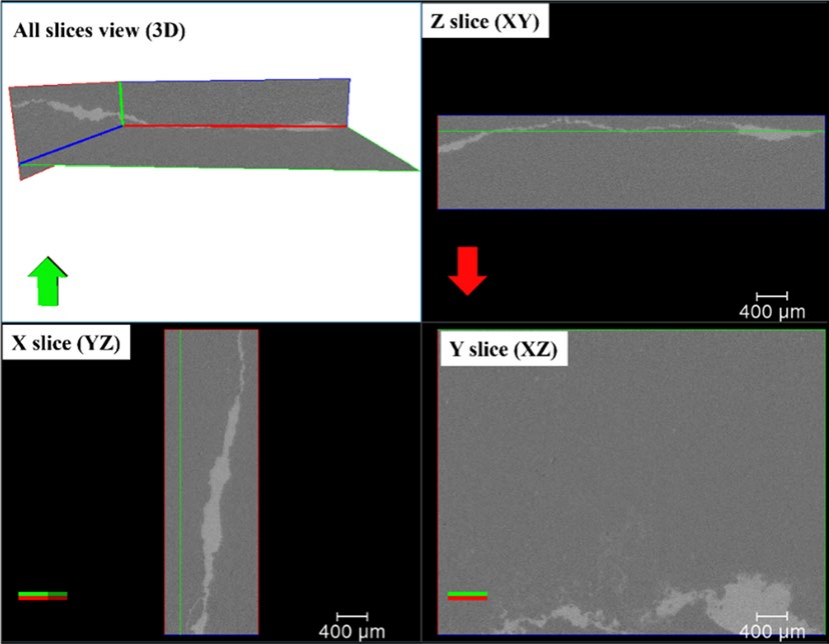
The raw greyscale scans are uploaded as 2D slices in each direction (*X, Y, Z*) as shown, to be pre-processed for segmentation and analysis. These orientations are important for aperture computations subsequently, and for identifying appropriate flow direction (in this case, the *Z* direction, viewed in *XY* cross section)

**Fig. 3:**
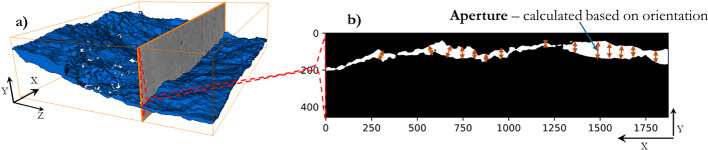
**a** Example of a 3D-rendered fracture void (blue) extracted from the surrounding rock matrix (grey slice) after image segmentation. **b** A 2D cross-sectional slice (binary image) showing fracture void in white and matrix in black (units are in pixels). The aperture at a given location is measured as the perpendicular distance between the opposing fracture walls. Note: *X* is the nominal flow direction

Image processing was performed using Thermo Fisher PerGeos 2021.1 software. The raw reconstructed volumes (Fig. 2) were first filtered using the anisotropic diffusion filter to reduce noise while preserving edges. Each image is then segmented to separate the fracture void from the surrounding rock matrix. The *interactive overlay threshold* tool was used to interactively place seed markers (void and matrix), after which the *marker-based watershed* segmentation option was used to grow these markers. Small, isolated artefacts and speckles were removed by applying an *axis connectivity* filter (retaining only the void clusters which corresponds to the main fracture). The result was a binary 3D image for each sample, with voxels classified as either fracture void (1) or rock matrix (0). Some of the image samples were slightly cropped to exclude artefacts and subtle branches, focusing on the single fracture. This explains why all sample dimensions are not the same (Table S2 of supporting information). From the binary volume, we digitally extracted the fracture void as a discrete object for analysis (Fig. 3a). Subsequent analyses were performed on this extracted void geometry using custom python code. A table of parameters typically used for the segmentation is provided in Table S1 in supporting information.

### Fracture Aperture Characterization

#### Aperture Definition and Measurement

The aperture *a* is a critical parameter for fracture characterisation. We define it as the local separation between the two fracture surfaces (or thickness of the void space). For each vertical column along the *Y*-direction, the uppermost and lowermost fracture voxels are identified, corresponding to opposing fracture walls. Binary masks of these wall voxels were generated. A Euclidean distance transform (EDT) was then applied separately to the top-wall and bottom-wall masks. At each fracture voxel, the local aperture was calculated as the sum of its distance to the nearest top wall and bottom wall voxel, multiplied by the physical voxel size (2.75 µm). This ensures that aperture values represent the separation between opposing walls rather than the distance from a single surface. Non-fracture voxels were assigned an aperture of zero.

To visualize the aperture fields, we generated 2D maps. A maximum intensity projection of the 3D aperture field is taken along the vertical (*Y*) axis, effectively collapsing the fracture aperture data onto an *X*–*Z* plane, coloured by the aperture value. This yields a map of aperture variations across the fracture plane.

#### Aperture Contact Area and Distribution

Regions where the fracture was completely closed or below the image resolution appear as zero-aperture in the void label of the segmented image (i.e. the opposing surfaces are in contact). We classified any voxel with an aperture less than one voxel (< 2.75 µm) as a contact point (zero aperture). By summing these zero-aperture voxels and normalizing by the fracture surface area, we computed the contact area percentage for each fracture, which quantifies how much of the fracture surface is in contact with opposing surface (or effectively closed).

The distribution and spatial variability of fracture aperture are key determinants of fluid conductivity through a fracture. Apertures in a single fracture can vary widely from point to point, leading to preferential flow channels where apertures are larger. This phenomenon of uneven flow is known as flow channelling (Tsang and Tsang 1989; Zimmerman and Bodvarsson 1996). This phenomenon shows the importance of understanding fracture type, aperture distribution, and heterogeneity when assessing permeability and flow dynamics. For each fracture, we quantified and visualized the aperture distribution across the entire fracture plane. The profiles provide a visualization of the aperture variation along the fracture length as well as the matedness of the surfaces.

#### Fracture Roughness Metrics

Roughness generally refers to the complexity of surface deviations from a reference plane. In a fracture context, rougher surfaces produce more variable apertures when the fracture is open. Because fracture roughness controls flow through different geometric facets, we use three complementary measures: one tracks how much the aperture varies, another shows at what scales that variability occurs, and a third captures how sharp or steep the surface asperities are. Together, these give a better picture than any single statistic could and include:

**Relative Roughness (*λ*):** From each aperture field we calculate key statistical measures: mean aperture *a*ₘ, standard deviation of aperture, and the coefficient of variation (CV = standard deviation/mean). The coefficient of variation can also be referred to as relative roughness (*λ*) for fractures, as it indicates the degree of deviation from a smooth fracture (for a homogenous aperture field, *λ* = 0). We use the term relative roughness *λ* for consistency. This is the only roughness metric based on aperture field and the other two are based on the surface profiles. To probe local behaviour, we subdivide the fracture domain into four subdomains and compute their statistics as well. Note that the number of subdivisions is random.

**Hurst Coefficient (H):** The Hurst coefficient is a statistical parameter that describes how surface height variations scale with length, commonly obtained via power spectral density (PSD) analysis of surface profiles. A self-affine fracture surface will have a Hurst exponent between 0 and 1, reflecting anisotropic scaling of roughness (Adler et al. 2012). We computed H by analysing the full set of one-dimensional profiles along both principal directions (*X* and *Z*) of each fracture surface. For each sample, we extracted all row-wise (*X* direction) and column-wise (*Z* direction) height profiles from the top and bottom surfaces and computed the power spectral density (PSD) of each individual profile using a Fourier-transform-based periodogram. This approach captures the full variability in roughness across the surface rather than compressing it into a single averaged representation.

The resulting PSDs were inspected to identify frequency ranges exhibiting power-law behaviour, where ${\mathrm{PSD}}\left( f \right) \propto f^{ - \beta }$. The spectral slope β was determined by fitting a linear model in log–log space within this range, and the Hurst exponent was calculated as *H* = (*β − *1)/2, consistent with theoretical expectations for self-affine 1D profiles extracted from 2D surfaces. This method allows us to compute a distribution of Hurst exponents for each surface and direction, from which we report the mean *H* value per direction (denoted *H*_top_ and *H*_bottom_), while also retaining information on the variability and directional anisotropy of surface roughness. This granular analysis, adapted from the approach by Bisdom et al. (2021) enables a more nuanced characterization of surface heterogeneity; lower *H* values correspond to rougher, more irregular surfaces (more high-frequency content), while higher *H* values indicate smoother surfaces with roughness confined to larger scales. Differences between *H* in *X* and *Z* directions could also reveal any surface anisotropy.

**Root Mean Square Slope (*Z***_***2***_**):** This parameter captures the average slope variability of the surface. For a discrete surface profile $y({x}_{i})$, this is given by

1 $$Z_{2} = \left[ {\frac{1}{{M\Delta x^{2} }}\mathop \sum \limits_{i = 1}^{M} (y_{i + 1} - y_{i} )^{2} } \right]^{0.5} ,$$

where *M* is the total number of measurements, and $\Delta x$ is the distance between two neighbouring measurements and ${y}_{i}$ is the surface height at coordinate ${x}_{i}$.

Higher *Z*_2_ values correspond to rougher surfaces with greater fluctuations in slope, while lower Z_2_ indicates a smoother profile. We computed *Z*_2_ for each fracture surface profile.

A commonly used roughness metric is the Joint Roughness Coefficient (JRC), an empirical roughness rating from 0 (smooth) to 20 (extremely rough) originally defined by Barton (1973). Li and Zhang (2015) have reported over 47 empirical relationships, most of which relate JRC with Z2. We note that these correlations were mostly derived at higher scale and resolution, so applying them to our microscale data yields values outside the original 0–20 range. This scale dependence suggests that although JRC remains a useful engineering metric, it may not be reliable for capturing roughness at the scale of our data for which extensive analysis is yet to be reported. It is thus not discussed in this study.

#### Spatial Correlation

Beyond local roughness, we assessed the spatial correlation of the aperture field for each fracture. Spatial correlation describes how aperture values at two locations are related as a function of their separation. A fracture with long-range spatial correlation will have regions of similar aperture persisting over some distance, whereas one with short correlation length will have aperture values change more randomly over short distances. We quantified spatial correlation using the geostatistical variogram. The variogram *γ*(*h*) is defined as half the mean squared difference between aperture values separated by a lag distance $h:$

2 $$\gamma \left( h \right) = { }\frac{1}{2N}\mathop \sum \limits_{i = 1}^{N} \left[ {a\left( {x_{i} } \right) - a\left( {x_{i} + h} \right)} \right]^{2}$$

where *N* is the number of data points separated by $h$, $a({x}_{i})$ and $a({x}_{i} + h)$ are the values of the variable in [mm] at location ${x}_{i}$ and ${x}_{i} + h$, respectively.

We computed experimental variograms (based on data) from the aperture data for each sample in both the *X* and *Z* directions of the fracture plane. A theoretical exponential model was then fitted to each variogram to characterize it. From the fitted model, we determined the correlation length *L*_c_ (range), defined as the lag distance at which the variogram reaches 95% of its sill (the sill being the plateau value representing the variance). The correlation length *L*_c_ represents the scale over which aperture values remain correlated. Beyond *L*_c_, aperture values become essentially uncorrelated. We performed variogram analysis using Python’s Geostatspy library (Pyrcz 2024). To improve statistical robustness, we calculated variograms on the full aperture field as well as on each of the four subdomains we created earlier.

The degree of aperture correlation is related to the matedness, a description of the overall match between fracture surfaces. Relative to tensile fractures, shear fractures are generally less mated due to displacement (Al-Fahmi et al. 2018; Cardona et al. 2021). Correlation and matedness thus influence the availability of flow paths as well as flow velocity. These make the correlation length metric important for comparing fractures. Furthermore, the correlation length influences pore occupancy and probability of trapping fluid in variable apertures (Jarsjoe and Destouni 1998).

Conceptually, the comparison employed in this study is agnostic to geological origin and instead targets the hydraulic imprint of mechanical character. In this data set, natural fractures are predominantly sheared and induced fractures predominantly tensile, but both modes can either arise in nature or be induced. Therefore, the geometric and hydraulic contrasts we look to quantify apply to whether fractures are sheared or tensile, regardless of origin.

### Flow Measurement and Modelling

We evaluated fracture flow using three complementary approaches: laboratory flow experiments, parallel plate modelling, and direct numerical simulations. The goal was to compare measured flow properties of our samples to model predictions and investigate how geometric complexity (roughness, heterogeneity) affects flow in the various fracture types.

#### Laboratory Core Flooding

We interpret the core-flood results by treating the system as a fracture-matrix ensemble. Because the matrix of the caprock has very low permeability (~ 10^–18^ m^2^), it was assumed that all flow is essentially through the fracture (Phillips 2022; Phillips et al. 2024). The fracture can thus be said to be connected in parallel to the matrix layers, with core permeability given as:

3 $$k_{{{\mathrm{core}}}} = { }\frac{{A_{m} k_{m} + A_{f} k_{f} }}{{A_{{{\mathrm{core}}}} }}$$

where ${k}_{\mathrm{core}} [{m}^{2}]$, ${k}_{m}$ and ${k}_{f }[{m}^{2}]$ denote the permeability of the core plug, matrix and fracture. ${A}_{\mathrm{core}} [{m}^{2}]$, ${A}_{m} [{m}^{2}]$ and ${A}_{f }[{m}^{2}]$ are cross-sectional areas of core, matrix and fracture, respectively. ${k}_{\mathrm{core}} [{m}^{2}]$ is calculated using Darcy’s law and ${k}_{m} [{m}^{2}]$ is assigned an analogue value. ${A}_{\mathrm{core}} = {\pi r}^{2}$*;* ${A}_{m} ={A}_{\mathrm{core}}-{V}_{f}/L$ where $r$ and *L* are the radius and length of the core, respectively. The fracture volume ${V}_{f}$ is obtained from analysis of the micro-CT images.

${A}_{f}$ is typically approximated by $w\cdot {a}_{m}$, where $w$ and ${a}_{m}$ are fracture width and mean mechanical aperture. For a real and rough fracture with variable apertures, however, ${A}_{f}$ cannot be reliably calculated and thus the exact flux through the fracture is unknown. The value of ${k}_{f}{A}_{f}$ can however be calculated from Eq. (3), as the transmissivity ${T}_{f}$ across the fracture width. As such, fracture flow is more suitably described by transmissivity ${T}_{f}$ rather than analytical permeability ${k}_{f}$*,* as it directly relates to flow capacity across the entire width. ${T}_{f}$ following Eq. (3) is given by:

4 $$T_{f} = { }k_{{{\mathrm{core}}}} A_{{{\mathrm{core}}}} - k_{m} A_{m} .$$

Note that ${T}_{f} [{m}^{4}]$ is considered for flow through a unit area (across the entire fracture width) rather than unit width which would consider a single aperture along the width.

An equivalent uniform aperture that would yield the observed flow under our test conditions, the hydraulic aperture ${a}_{exp}$, considering ${A}_{f}=w\cdot {a}_{m}$ and $T={k}_{f}{A}_{f}$, is given by

5 $$a_{{{\mathrm{exp}}}} = { }\frac{{T_{f} }}{{k_{f} w}}$$

The hydraulic aperture provides a convenient way to compare the real fracture (with roughness and variable aperture) prediction of flow, to an ideal smooth fracture with aperture ${a}_{m}$, and obtain insight into effective conductivity. In parallel, we used the aperture fields to perform analytical flow predictions and direct numerical simulations on raw images for comparison.

#### Cubic Law Model

Using the mean mechanical aperture (*a*_m_) obtained from the image data, we applied the cubic law formula for a fracture:

6 $$T_{f} = { }\frac{{wa_{m}^{3} }}{12}$$

This represents the transmissivity assuming that the fracture is a smooth parallel plate of uniform aperture equal to the mean.

#### Direct Numerical Simulation

We employed direct numerical simulation (DNS) using the open-source computational fluid dynamics (CFD) toolbox GeoChemFoam (Maes and Menke 2021), an extension of OpenFOAM designed to simulate complex flow processes in porous media. This approach directly resolves the governing flow equations within the actual 3D fracture geometry, thereby capturing the effects of surface roughness and aperture variability without resorting to simplified geometric assumptions.

The simulations employed the steady-state incompressible Navier–Stokes equations as implemented in OpenFOAM’s simpleFoam solver:

7 $$\rho \left( {u.\nabla } \right)u = - \nabla p + \mu \nabla^{2} u$$

8 $$\nabla .u = 0$$

where $\rho$ denotes the density of the fluid, $u$ the velocity vector field, $p$ the pressure field, and $\mu$ the dynamic viscosity of the fluid. The convective term $(u\cdot \nabla )u$ was fully retained, ensuring that inertial effects are resolved if present. However, for the conditions investigated, the Reynolds number (Re) was extremely low (Re = 3 × 10^–5^), placing the flow within the creeping-flow regime and likely towards the Stokes limit. Previous studies have investigated the applicability of Re for permeability estimations in rough fractures as well as compared Navier–Stokes to Stokes flow (Brown et al. 1995; Brush and Thomson 2003; Lee et al. 2014; Mourzenko et al. 1995; Zimmerman et al. 1991; Zimmerman and Yeo 2000). For this work, the immediate differences in account for non-linearity is not considered.

Upon achieving a converged flow field, the permeability ${k}_{\mathrm{sim}}$ of the fracture is calculated using Darcy’s law,

9 $$k = \frac{{Q{\upmu }L}}{A\Delta P}$$

Pressure equations utilized geometric-algebraic multigrid (GAMG), and velocity equations employed a smooth solver (Gauss–Seidel), both set with convergence tolerances of 10^–6^.

Although permeability calculations ultimately use a single representative cross-sectional area (typically the inlet area), local variations in aperture and face areas throughout the computational domain are inherently captured by the cell-based discretization. Each cell's solver accounts for the local geometry, ensuring accurate representation of spatial heterogeneity in flow conditions. Consequently, the local flow velocities and pressure gradients adapt to these geometrical features, influencing the overall pressure drop required to maintain the imposed flow rate. This approach allows for an adequate assessment of how aperture heterogeneity impacts permeability, without resorting to empirical correlations or simplified geometric assumptions. Further description of the approach is found in supporting information.

To facilitate direct comparison between experimental results and numerical outcomes, the equivalent hydraulic aperture from simulation (${a}_{\mathrm{sim}}$) was computed by rearranging the cubic law equation to yield the aperture corresponding to the observed pressure drop and flow rate. By comparing the mechanical aperture (${a}_{m}$), experimental hydraulic aperture (${a}_{\mathrm{exp}}$) and DNS-derived aperture (${a}_{\mathrm{sim}}$), we quantified deviations from idealized flow predictions and assessed differences attributable to fracture geometry and permeability estimation method. Importantly, unlike comparative studies using unrelated laboratory-induced fractures and field samples, our study uses same lithology. All five fractures come from the same formation and experienced the same diagenetic history, ensuring differences in flow properties reflect nature of fracture rather than rock properties alone.

## Results and Discussion

### Fracture Aperture Characteristics

**Fig. 4:**
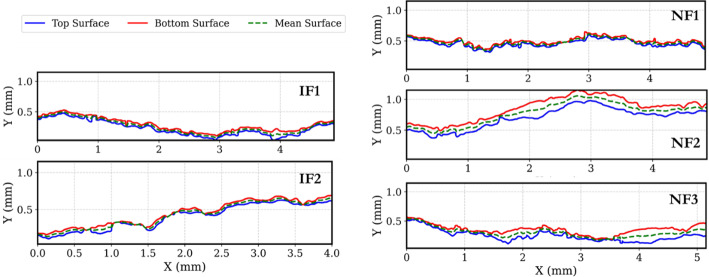
2D *XY* slices showing the surface profiles for each sample. This also illustrates the existing degrees of matedness and the orientations. The mean surface is the average of the top and bottom surfaces. Note that these slices are randomly selected and represent only a small cross section of the highly variable surfaces

**Fig. 5:**
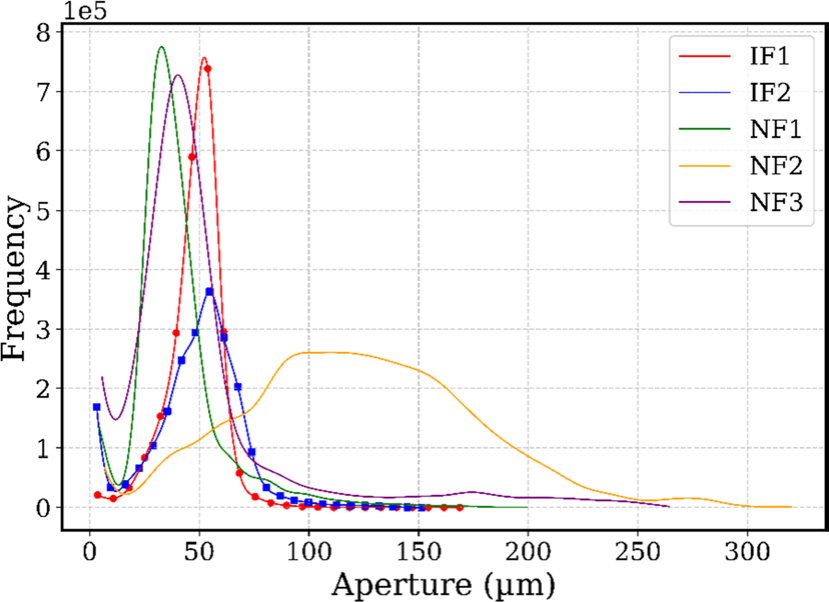
Histograms of aperture distributions for all five fractures (aperture in micrometres on the *x*-axis). All samples show positively skewed distributions. The natural fractures (NF1–NF3) exhibit longer high-aperture tails (more extreme large-aperture occurrences) compared to the induced fractures with markers (IF1–IF2), indicating a slight increase in skewness for the NFs

**Fig. 6:**
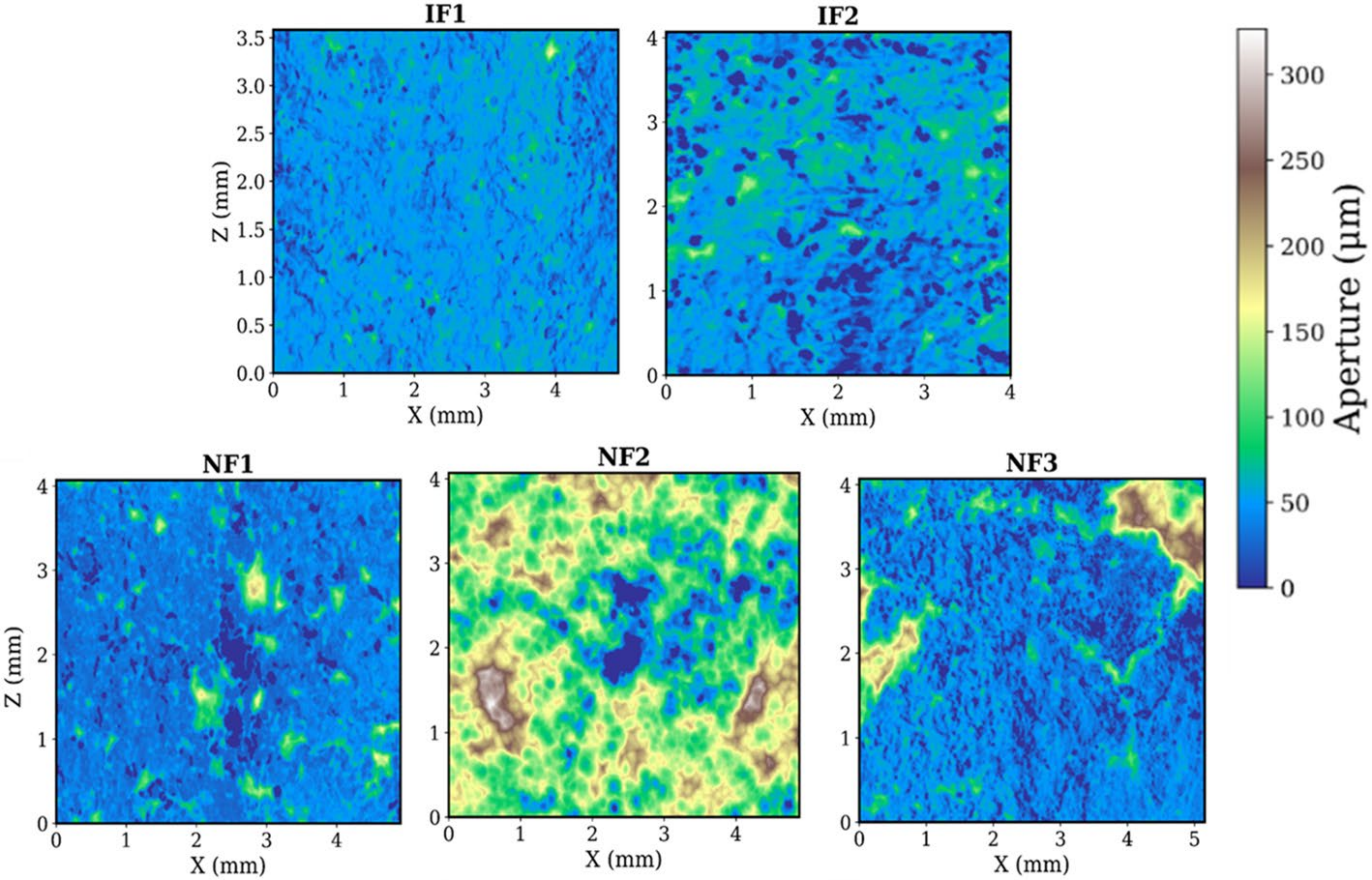
Colour maps of aperture across the fracture plane (projected in the *X*–*Z* plane) for each sample. The induced fractures (IF1, IF2) show a relatively regular arrangement of aperture and contact spots across the plane, whereas the natural fractures (NF1–NF3) display irregular, patchy patterns with localized zones of very large aperture separated by extensive contact areas. These maps visually emphasize the greater heterogeneity in the natural fractures

The induced fractures (IFs), show a slightly higher match between opposing fracture walls (matedness), compared to the natural fractures NFs (Fig. 4). Figure 5 presents the aperture distribution (histograms) for all fractures, and Fig. 6 shows aperture maps for visual comparison.

The histogram of the apertures (Fig. 5) show positively skewed distributions for all fractures, with NFs exhibiting longer tails due to localized regions of larger apertures. This similarity in distribution between types aligns with previous studies conducted mainly on granites (Table 1) which found that tensile and shear fractures can have the same distributions, and that distributions are rather influenced by local heterogeneity than by fracture type alone (Méheust and Schmittbuhl 2001). In fact, the calculated mean mechanical aperture (*a*ₘ) for all samples falls within the range of 49–146 µm, which also have overlapping values for contact area percentage. This suggests that while there may be slight differences, this cannot be exactly captured by non-spatial statistics, as they overlap for all fractures. A summary of the global aperture statistics is provided in Table 2. The histogram of NF2 is evidently different from the other samples (Fig. 5), with the highest standard deviation but also significantly higher mean, which lowers its statistical variability (Table 1). This just demonstrates the uniqueness of single fractures.

**Table 2:** Summary of statistical metrics that describe the spread and variability in the fracture apertures and surfaces. 'Top' and 'Bottom' for the Hurst coefficient (H) refer to the fracture surfaces on each side of the fracture opening

	IF1	IF2	NF1	NF2	NF3
Mean aperture, *a*_m_ (µm)	48.35	47.68	39.91	121.58	51.41
Max. aperture (µm)	172.35	154.49	203.65	326.91	269.72
Standard deviation, SD (µm)	12.21	21.55	21.54	54.38	43.49
Relative roughness, *λ*	0.25	0.45	0.54	0.45	0.85
Contact ratio (%)	0.65	6.91	4.27	2.13	5.12
Correlation length *X*, *L*_c__x (mm)	0.22	0.289	0.33	1.4	1.47
Correlation length *Z*, *L*_c__z (mm)	0.165	0.35	0.42	0.75	0.81
*H*_top_x_	0.63	0.64	0.72	0.46	0.72
*H*_top_z_	0.64	0.46	0.53	0.48	0.43
*H*_bot_x_	0.63	0.73	0.78	0.50	0.69
*H*_bot_z_	0.67	0.44	0.63	0.48	0.50
*Z*_2_	0.89	0.87	1.02	0.83	2.08

Figure 6 however shows some evidence of distinct differences in spatial behaviour. The IFs have more regular distribution of contact and large aperture regions, compared to the NFs which show more irregular and random distribution.

The large, continuous yet localized aperture and contact regions in the NFs thus suggests a more heterogenous aperture field that makes flow more complex. NF2 with its rather different distribution, still conforms to the spatial behaviour shown by the other NFs. We attempt to capture this spatial behaviour in the next sections on heterogeneity and spatial correlation.

### Fracture Roughness and Heterogeneity

From Table 2, we see that relative roughness (*λ*) is higher for the natural fractures. IF1 and IF2 have *λ* of 0.25 and 0.45 (indicating homogeneous aperture fields, especially IF1), whereas the NFs show *λ* values ranging between 0.45 and 0.85 (NF3 being the highest, very heterogeneous). In fact, the IFs have both the highest and lowest contact area percentages (IF2—6.91%, IF1—0.65%), but from the aperture maps, the contact points are more uniformly arranged with narrower spread than the NFs. This explains the more heterogeneous behaviour in the latter set. It is worth noting that the interpretation of *λ* is meant to provide relative understanding of overall behaviour (perfectly homogenous fields have *λ* = 0) as all fractures have a degree of heterogeneity. The heterogeneity of the aperture field is influenced by the variability on the fracture surfaces, which are evaluated using the power spectral density approach.

**Fig. 7:**
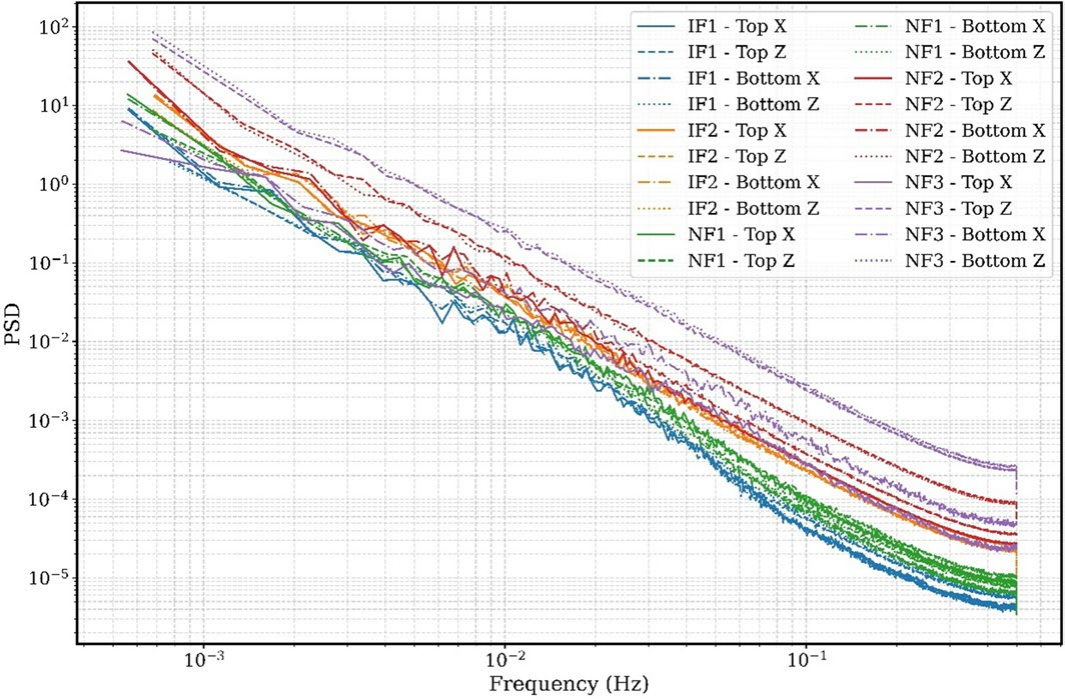
Power spectral density (PSD) as a function of spatial frequency for fracture surface profiles of each sample (profiles taken in *X* and *Z* directions, for both top and bottom surfaces). All samples exhibit a roughly linear negative slope, indicating self-affine roughness. The similar slopes (and thus Hurst exponents) across samples suggest comparable scaling properties of roughness for both natural and induced fractures. NFs generally show larger power differences between their *X* and *Y* surfaces at higher frequency, which suggests potential anisotropy

Figure 7 illustrates the power spectral density (PSD) behaviour across samples. The curves for all samples have a negative slope, meaning that the amplitude of surface height variation generally decreases at higher frequencies or finer scales. The Hurst coefficient (H), calculated from the slope of the curves, reports values < 1 for all samples (Table 2) and tends to be generally higher in *X* than in *Z* directions. This provides an indication of anisotropy, with a generally higher roughness profile in *Z* direction. There is however little indication that this behaviour can be attributed to fracture type. The PSD plots for each fracture are found in supporting material.

The RMS slope (*Z*_2_) values reflect some differences: NF3 has a much higher *Z*_2_ value of 2.08 compared to the other fractures, consistent with it being the roughest fracture surface by visual inspection. The *Z*_2_ values for NF1 and NF2 are 0.8–1.0, comparable to the IFs (0.87–0.89). As a result, NF1 and NF2 have only slightly higher average slopes compared to the IFs, while NF3 is an outlier with very steep micro-roughness.

Our results suggest that surface roughness metrics alone are insufficient to clearly distinguish between fracture types. This aligns with prior observations by Brown and Scholz (1985b) who found that surface roughness statistics can be similar for different types of fractures (even a natural shear vs. a tensile fracture in siltstone might show similar PSD curves). Several studies have demonstrated the impact of roughness on hydraulic transmissivity in single fractures. However, Méheust and Schmittbuhl (2001) noted that fractures with similar roughness statistics could exhibit different flow behaviours depending on fracture geometry and spatial correlation. The variability, controlled by spatial correlation of the apertures, is highlighted as a key differentiating factor. Earlier research by Brown and Scholz (1985a) also highlighted spatial correlation beyond fractal dimensions as a crucial parameter influencing flow variability, emphasizing the importance of assessing roughness in combination with aperture correlation analysis.

### Spatial Correlation

**Fig. 8:**
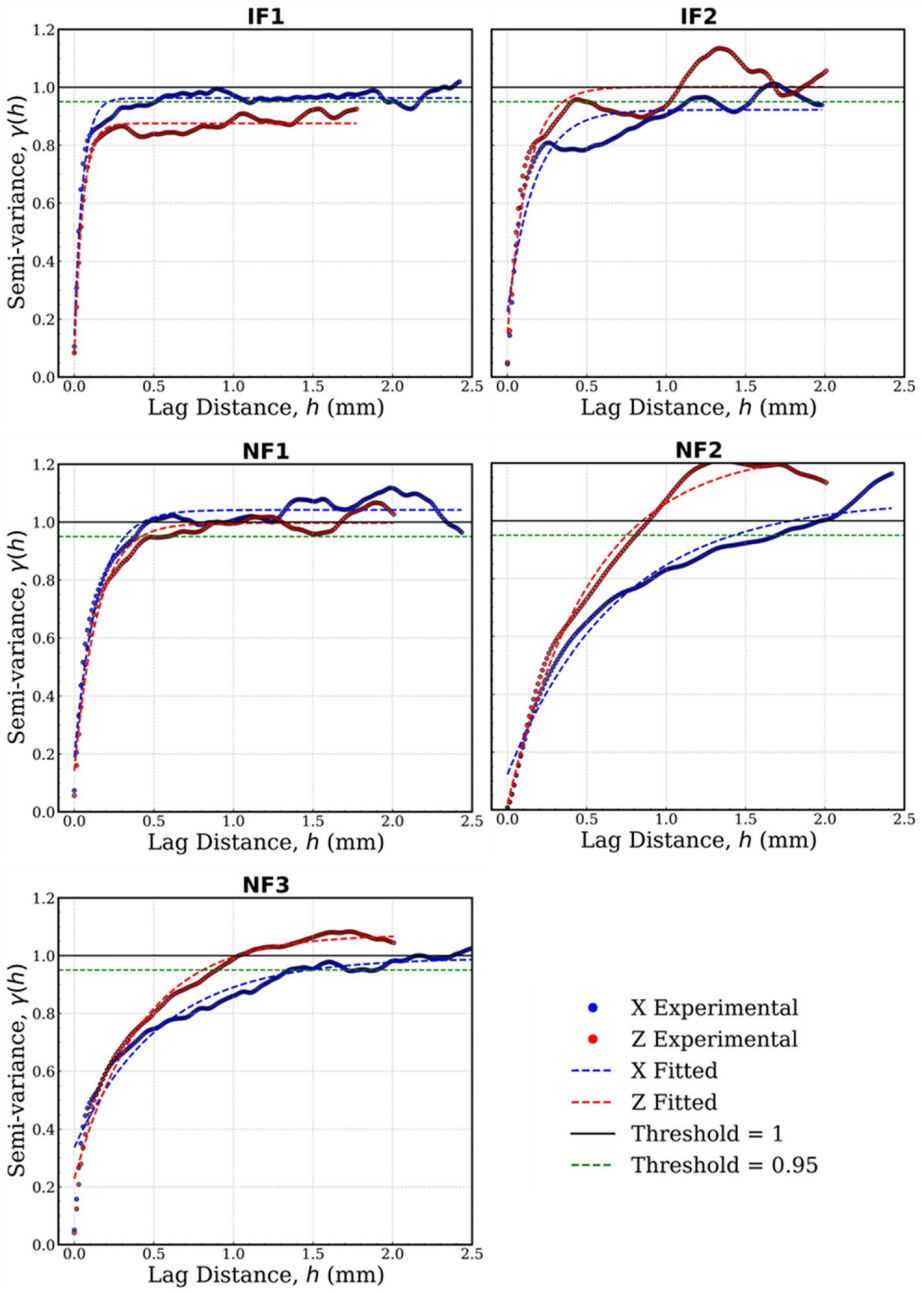
Variogram of each sample fitted with an exponential model. The correlation length is the lag distance at which the variogram reaches 95% of the sill or levels off. The sill has been normalized to 1

The spatial correlation of the aperture field is quantified, using the geostatistical variograms (Fig. 8). The correlation lengths (*L*_c_, Table 2) show low values for IF1 and IF2 of 0.2–0.3 mm, whereas the natural fractures show higher values of 0.5–1.5 mm. The data suggest that NFs, despite their variability, have segments that maintain a certain aperture over longer distances, possibly reflecting offsets from shearing. In contrast, the IFs are more uniformly rough at small scale and alternate between open and closed more frequently. This results in shorter correlation lengths.

**Fig. 9:**
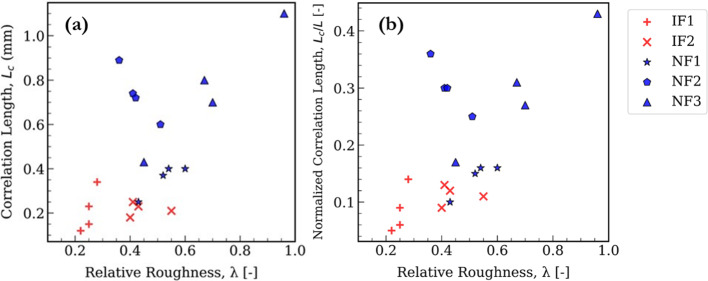
Relative roughness *λ* plotted against aperture correlation length *L*_c_. **a** *λ* versus *L*_c_ obtained for both *X* and *Y* directions. *L*_c_ generally increases with *λ*; IFs generally plot into low *λ*–low *L*_c_ area, **b** *L*_c_ is normalized against *L*, allowing comparison across scales

Figure 9 shows a possible correlation between relative roughness (*λ*) and correlation length (*L*_c_) across the samples. Such correlation has previously been reported on the centimetre scale by Hakami (1995), using a collection of experimental studies on granites and sandstones, with both shear and tensile fractures. Essentially, the NFs plot in a high-*λ*, high-*L*_c_, whereas IFs in a low-*λ*, low-*L*_c_ regions (Fig. 9). The same study also suggested the use of these metrics because they were less sensitive to stress changes than other non-spatial metrics. The trend suggests that more heterogeneous fractures also have larger coherent aperture structures (perhaps large undulations that create both large open voids and large closed zones). While NF2 shows expected behaviour with *L*_c_, its lower local *λ* values causes it to slightly oppose the general trend at local scale. The unique aperture distribution of NF2 has previously been noted. It is important to note that our image samples are small (a few mm), so these correlation lengths are also small in absolute values. Normalizing *L*_c_ by the fracture length *L* did not change the relative ordering; NFs still had higher *L*_c_/*L* ratios than IFs.

The trends for NFs (Fig. 9), contrasts with findings from previous studies on natural (tensile) fractures measured on coarser-grained and more mechanically competent granites (Table 1). This difference in trend for the natural fractures (in this study versus the other studies) is attributed to the nature of fracture formation, specifically whether a fracture is sheared or tensile. Sheared fractures (such as our samples) exhibit wall mismatches due to displacement, leading to highly variable aperture distributions. In contrast, tensile fractures, particularly those that are well-mated, tend to have more uniform aperture distributions with lower spatial variability. Studies by Gentier (1990) and Iwano and Einstein (1993) on natural tensile fractures support this observation, demonstrating that well-mated fractures exhibit less spatial heterogeneity compared to sheared, unmated fractures. Therefore, a natural fracture that is not sheared and that remains well-mated would exhibit spatial characteristics like an induced tensile fracture, reinforcing the role of fracture genesis in controlling aperture variability and flow behaviour.

In summary, the geometric characterization shows that while average aperture and some roughness measures may not differ substantially between induced and natural fractures, the spatial aspects (heterogeneity, contact area, correlation length) do differ. Natural shear fractures exhibit a combination of high heterogeneity and longer-range spatial structure, whereas induced tensile fractures are more homogeneous and lack large-scale aperture streaks. The NFs will thus potentially have complex and tortuous flow paths than the IFs.

### Hydraulic Properties

**Fig. 10:**
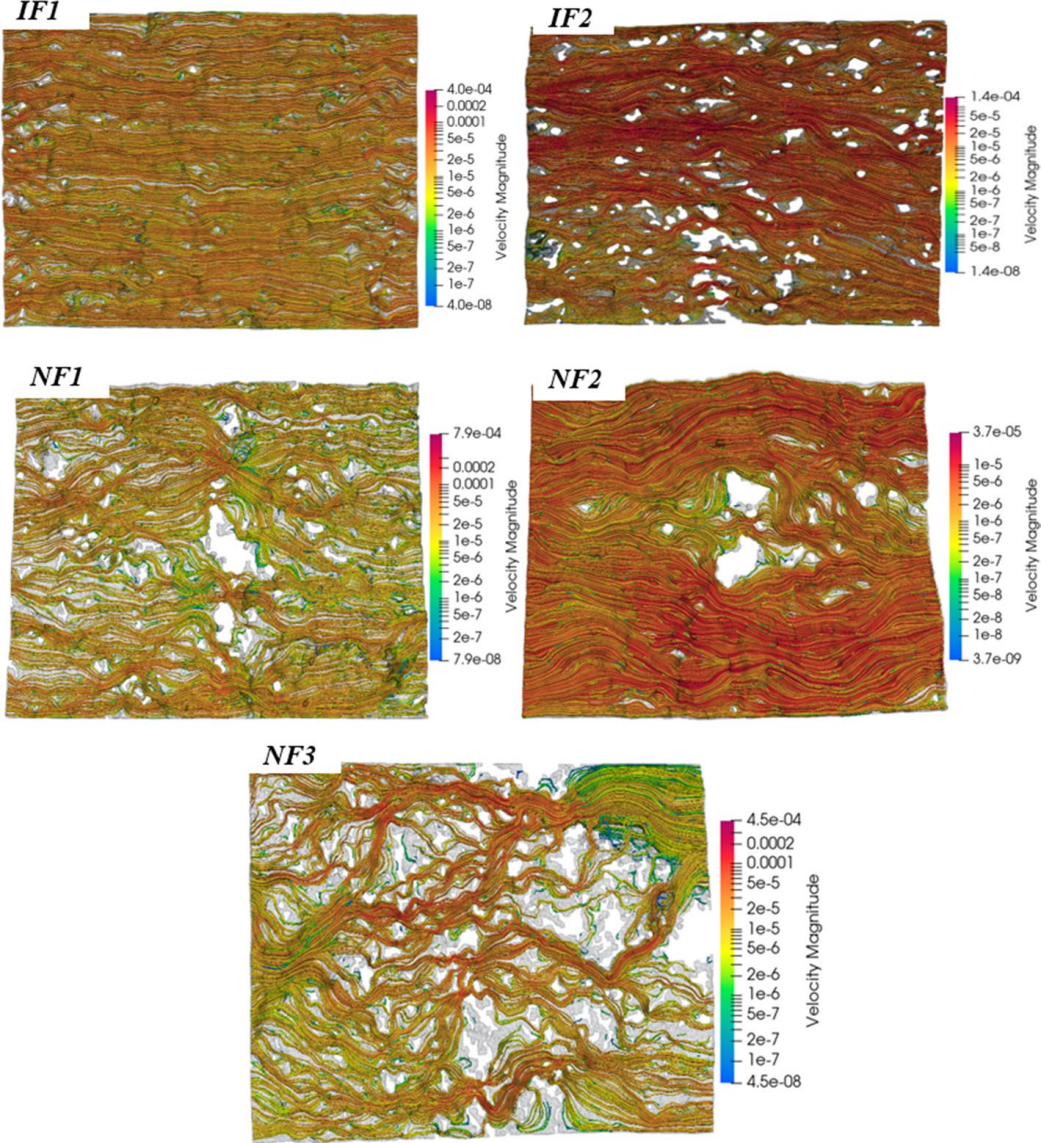
Streamlines of the magnitude (m/s) of single-phase velocity field for each fracture, obtained from direct numerical simulation on the segmented images

The impact of the geometric differences becomes evident when analysing fluid flow results. Streamlines of the velocity fields at the end of simulation provide some illustration of this effect (Fig. 10). The transmissivity for each fracture was measured and computed from experiments, and permeability from direct numerical simulations (Table 3).

**Table 3:** Transmissivity and permeability values for IFs and NFs. These are measured from experiment (exp), calculated using the cubic law (CL) model and direct numerical simulations using GeoChemFoam. The corresponding hydraulic apertures are also shown

		IF1	IF2	NF1	NF2	NF3
Mechanical aperture *a*_m_ (µm)	Arithmetic mean	48.40	47.70	39.90	122.00	51.40
Core plug transmissivity, *T*_core_ (m^4^)	Experiment	4.4E-18	8.1E-19	2.7E-19	7.0E-19	2.4E-19
Fracture transmissivity, *T* (m^4^)	Experiment, *T*_exp_	4.43E-18	8.12E-19	2.71E-19	6.96E-19	2.44E-19
	Cubic Law, *T*_CL_	3.37E-17	3.68E-17	2.16E-17	6.10E-16	4.61E-17
Fracture permeability, *k* (m^2^)	Cubic Law, *k*_CL_	1.95E-10	1.89E-10	1.33E-10	1.23E-09	2.20E-10
	Numerical simulation, *k*_sim_	1.77E-11	9.52E-12	4.18E-12	2.01E-11	3.04E-12
Hydraulic aperture, *a* (µm)	Experiment (*a*_exp_)	24.60	13.40	9.27	12.70	8.95
	Simulation (*a*_sim_)	14.58	10.69	7.08	15.83	6.04
Ratios	*a*_exp_*/a*_sim_	1.69	1.25	1.31	0.80	1.48
	*a*_exp_*/a*_m_	0.51	0.28	0.23	0.10	0.17
	*a*_sim_*/a*_m_	0.30	0.22	0.18	0.24	0.12

The measured transmissivities (*T*_exp_) indicate that the two induced fractures (IF1, IF2) are more transmissive than any of the NFs. Fracture and core plug transmissivities are equal, which validates our assumption that flow is constrained to the fractures. The equivalent fracture permeabilities, estimated using hydraulic apertures, are within 10^–11^–10^–12^ m^2^. When compared to the intact matrix permeability, about 10^–17^–10^–18^ m^2^ (Phillips 2022; Phillips et al. 2024), these values are several orders of magnitude higher and confirm that even a single fracture can dramatically increase flow.

**Fig. 11:**
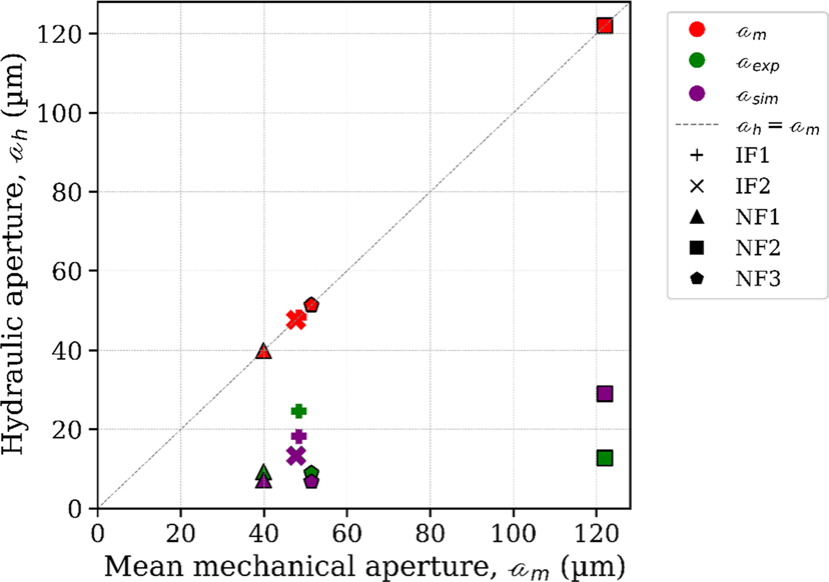
The mean mechanical aperture is plotted against the hydraulic apertures *a*_*h*_, computed from aperture measurement (*a*_*m*_), flow experiment (*a*_exp_) and numerical simulation (*a*_sim_). All samples show deviations from idealized parallel-plate approach (*a*_*h*_ = *a*_*m*_), demonstrating that every fracture’s effective flow aperture is lower than its mean physical aperture due to roughness and contact areas. Notably, NF2 despite its much larger mean aperture, plots similarly to the others in terms of hydraulic aperture, illustrating how fracture heterogeneity reduces flow capacity

The CL-based fracture permeabilities using mean mechanical aperture are one—to—two orders of magnitude higher than the simulated and equivalent measured permeabilities for both fracture types. This is however consistent with the suggestion that the model (cubic law) can approximate flow to within 2 orders of magnitude for all reasonable roughnesses (Brown 1987). But it is important to illustrate how this applies to different fracture types. Figure 11 shows the mean mechanical aperture *a*_m_ plotted against measured (*a*_exp_) and simulated (*a*_sim_) hydraulic apertures.

The measured hydraulic aperture *a*_exp_ has a range of 13–25 μm for IFs and 9–13 μm for NFs. Interestingly for instance, despite the large mean aperture of NF2 (122 μm), its measured hydraulic aperture is approximately one order of magnitude smaller or only 10.6% of *a*_m_ (13 μm). This emphasizes how much of its cross section is effectively non-conductive. Meanwhile, *a*_exp_ of IF1 (25 μm) is about half its mean aperture (48 μm), suggesting that about half of its fracture opening was effectively contributing to flow, a far better efficiency compared to NF2. This efficiency for all fractures is captured by the ratios *a*_exp_/*a*_*m*_ and *a*_sim_/*a*_*m*_ (Table 3) which are all < 1, indicating that the geometries of all fractures are flow-inhibiting (Méheust and Schmittbuhl 2001), although more pronounced for the NFs. They further reveal that only about 30–50% (for IFs) and 10–25% (for NFs) of the mean mechanical aperture effectively contributes to flow.

This result is due to aperture heterogeneity and would ultimately suggest that natural shear fractures are less efficient at transmitting flow relative to their mean aperture than induced tensile fractures are. The resulting effect is that flow may be overestimated when using tensile fractures. In this study however, the equivalent permeabilities for both IFs and NFs are either on the same or within an order of magnitude. This suitably reflects the fact that although the degrees may differ, all the fractures have heterogeneous aperture fields which influence flow. We infer that this similarity in resulting impact is influenced by the fine grain size of the intact rock as demonstrated by Iwano and Einstein (1993), and the differential impact may be more significant for fractures in coarser-grained rocks.

The results provide an evidence‐based comparison of IFs and NFs that offers insight into the influence of aperture geometry on fracture flow. The flow-impeding effects of heterogeneity and shear have been documented by Zimmerman and Bodvarsson (1996) and further modelled by Barton et al. (1985). The less dramatic difference in hydraulic apertures suggests that rough fractures of very different geometry can yield similar bulk flow apertures, and that all fractures may not hydraulically be the same, even with identical non-spatial characteristics.

### Implications, Scale and Limitations for Comparison of Natural and Induced Fractures

Our approach is deliberately local, with pore-scale simulations and aperture statistics derived from small μCT windows, while the core-flooding experiments integrate flow over the full core plug. The good agreement between window-based transmissivities and whole-plug measurements indicates that these sub-volumes are reasonably representative at the plug scale and supports the use of high-resolution subsets to characterise fracture behaviour at core scale.

The results show that induced tensile fractures can mimic some characteristics of natural shear fractures, but they are more efficient conduits for flow. When relying on tensile fractures to estimate shear fracture behaviour, permeabilities could therefore be overestimated unless adjustments are made. Because the study is intrinsically comparative (all fractures from the same caprock under identical conditions), these *relative* contrasts between shear and tensile fractures are expected to be more robust than any individual permeability value, even if absolute values shift under different stress or temperature conditions.

For this specific fine-grained caprock, the difference in overall flow character between fracture types is limited: the difference in effective hydraulic behaviour between fracture types is much smaller than their collective deviation from homogeneous flow (parallel-plate model using mean mechanical aperture). A key outcome is that the method of computing permeability and flow introduces at least as much uncertainty as whether the fracture is tensile or sheared. This is encouraging because fractures are easier to generate and study in the laboratory; they can serve as an upper-bound analogue for the permeability of naturally-occurring fractures if properly interpreted and calibrated (for example, using ratios between mechanical and hydraulic apertures).

From a modelling perspective, GeoChemFoam is used as a pore- and fracture-scale CFD tool, not as a reservoir simulator. It resolves flow directly on segmented μCT geometries, and propagating the resulting properties to reservoir scale requires explicit upscaling choices (discrete-fracture, dual-permeability or network models). In all such frameworks, the relevant flow parameters ultimately originate from pore- and core-scale measurements of the type presented here.

Finally, the fractures analysed are few, drawn from a single fine-grained claystone caprock, imaged over small windows and tested under one effective normal stress at room temperature, without thermal or chemical evolution. Our findings therefore represent an in-depth comparative case study rather than a statistical survey, and care should be taken when extrapolating to different lithologies and scales, where coarser or more brittle rocks may exhibit larger contrasts between shear and tensile fractures.

## Conclusions

We compared three natural shear fractures and two induced tensile fractures in a claystone caprock using high-resolution imaging, core-flooding and direct numerical simulations. Although they share similar non-spatial statistics, natural shear fractures exhibit more heterogeneous and spatially correlated aperture fields than induced tensile fractures. Yet, the two groups show overlapping equivalent permeabilities, while all samples deviate strongly from homogeneous parallel-plate predictions.

Induced tensile fractures can therefore serve as upper-bound proxies for natural shear fractures in same-lithology caprocks, provided that their higher flow efficiency is explicitly corrected for (e.g. via calibrated relationships between mechanical and hydraulic apertures). Our results also show that uncertainty introduced by the choice of permeability estimation method is at least as large as that associated with fracture type, highlighting the need to quantify methodological bias when interpreting fracture flow.

These insights help constrain fracture transmissivity in caprock integrity assessments. Incorporating fracture genesis, spatial heterogeneity and calibrated hydraulic properties into fracture-network models should improve predictions of leakage risk and long-term containment in CO₂ and hydrogen storage or waste disposal projects.

## Supplementary Information

Below is the link to the electronic supplementary material.

Supplementary file1 (DOCX 4635 KB)

## Data Availability

The raw reconstructed scans of the scans reported in this paper are publicly available online at [10.6084/m9.figshare.30149215] (https://www.figshare.com/account/home) They are provided in *.tiff* format so interested users can import directly to their image analysis software of choice and visualise/analyse all slices or as a 3D volume. Custom python scripts used for characterisation will be uploaded to the same portal. Other processed data can be requested directly from the authors. The flow simulation scripts are open access with full description and tutorials on [GeoChemFoam.GitHub] (https://www.github.com/GeoChemFoam).
