# Supplementary material for: Analysis and Comparison of Natural Shear and Induced Tensile Fractures for Caprock Leakage Assessment
Source: Transp Porous Media. 2026 Jan 14;153(2):29. doi: 10.1007/s11242-025-02283-0 (PMC12804337; doi:10.1007/s11242-025-02283-0)
Supplement: Supplementary file 1 — Supplementary file1 (DOCX 4635 KB) [file 11242_2025_2283_MOESM1_ESM.docx]

*Water Resources Research*

Supporting Information for

**Analysis and Comparison of Natural Shear and Induced Tensile Fractures for Caprock Leakage Assessment**

Sahyuo Achuo Dze^1*^, Tomos Phillips^1, 3^, Reza Najafi-Silab^2^, Sarah Perez^1^, Tom Bultreys^3^, Vladimir Novak^4^, Christian M. Schlepütz^4^, Veerle Cnudde^3,5^, Florian Doster^2^, Kamaljit Singh^2^, Kevin Bisdom^6^, Andreas Busch^1^

^1^Lyell Centre, Heriot-Watt University, Edinburgh, UK

^2^Institute of GeoEnergy Engineering, Heriot-Watt University, Edinburgh, UK

^3^Centre for X-ray Tomography (UGCT), Ghent University, Ghent, Belgium

^4^Swiss Light Source, Paul Scherrer Institute, Villigen, Switzerland

^5^ Department of Earth Sciences, Utrecht University, Utrecht, The Netherlands

^6^Shell Global Solutions International BV, The Hague, NL

Corresponding author: [sba2002@hw.ac.uk](mailto:sba2002@hw.ac.uk)

Contents of this file

Text S1 to S5

Figures S1 to S7

Tables S1 to S3

Introduction

This Supporting Information document provides further context to the main article for the experimental datasets, processing steps, and computational workflows used in this study. The primary data comprise high-resolution synchrotron micro-CT images of natural shear and induced tensile fractures in clay-rich caprock samples. All image files are stored in 3D TIFF format and include both raw greyscale reconstructions and segmented binary masks for each fracture. Key segmentation parameters and representative image slices are provided to illustrate the preprocessing and fracture extraction procedure.

A series of custom Python scripts were used for data segmentation, fracture wall identification, aperture and surface profile analysis, and statistical characterization of fracture geometry. Processed datasets include aperture fields, power spectral density (PSD) curves, and derived mesh files for computational fluid dynamics (CFD) simulations. The mesh generation process and simulation approach are also included.

Known imperfections in the data mainly arise from imaging artefacts, partial volume effects at fracture edges, and the subjectivity inherent in segmentation threshold selection. All supporting scripts will be provided and additional data can be provided upon request to ensure reproducibility.

**Text S1: Raw data and Segmentation**

To illustrate data setup and segmentation process, Figure S1 (also found in the main article) shows representative slices from a reconstructed synchrotron CT scan prior to segmentation. Some representative parameters used for segmentation and highlighted in Table S1. Note that this is a user-subjective process and can be tuned to suit context. We focused on capturing the through-going single fracture in this study.


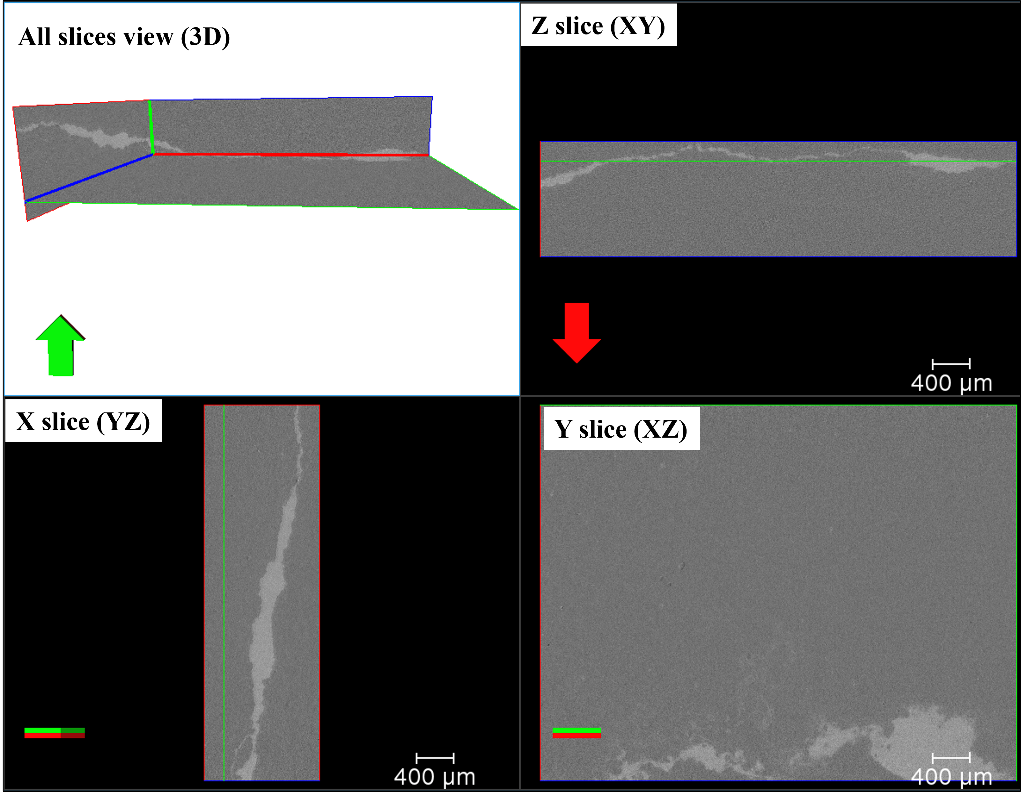


Figure S1: The raw greyscale scans are uploaded as 2D slices in each direction (X, Y, Z) as shown, to be pre-processed for segmentation and analysis. These orientations are important for aperture computations subsequently, and for identifying appropriate flow direction (in this case, the Z direction, viewed in XY cross-section)

Table S1: Parameters for segmentation (Case: NF3). These parameters provide the seed markers which the Watershed algorithm ‘populates’. An additional but optional step was used after segmenting called ‘Interactive top Hat’ with kernelSize = 12 and threshold between 2716 and 15004. This is meant to ‘top up’ void space believed to have been missed by watershed.

| Anisotropic diffusion | | | |
| --- | --- | --- | --- |
| Filter | 9 |  |  |
| Threshold | 18367.4 |  |  |
| Iterations | 55 |  |  |
| partitionSize | 10.85 |  |  |
|  |  |  |  |
| Interactive Threshold Overlay | | | |
|  | Label | Min | Max |
| Threshold value | Void | -65198 | -33222 |
|  | Matrix | -32308 | 11545 |
|  |  |  |  |
| Axis Connectivity | | | |
| Neighbourhood | 2 |  |  |
| Orientation | 2 |  |  |

Table S2: Key sample data from experiments and imaging.

|  | **sample_ID** | IF1 | IF2 | NF1 | NF2 | NF3 |
| --- | --- | --- | --- | --- | --- | --- |
| **Core measurements** | **Length (mm)** | 18 | 18 | 18 | 18 | 18 |
|  | **Diameter (mm)** | 6 | 6 | 6 | 6 | 6 |
|  | **Pressure drop (kPa)** | 1.1 | 6 | 18 | 7 | 20 |
|  | **Confining pressure (kPa)** | 45 | 45 | 45 | 45 | 45 |
|  | **Pore pressure (kPa)** | 20 | 20 | 20 | 20 | 20 |
| **Image Dimensions (pixels)** | **Length (X)** | 1777 | 1455 | 1786 | 1775 | 1873 |
|  | **Width (Z)** | 1303 | 1480 | 1480 | 1480 | 1480 |
|  | **Height (Y)** | 226 | 314 | 361 | 467 | 457 |
| **Image Dimensions (mm)** | **Length (X)** | 4.89 | 4.00 | 4.91 | 4.88 | 5.15 |
|  | **Width (Z)** | 3.58 | 4.07 | 4.07 | 4.07 | 4.07 |
|  | **Height (Y)** | 0.62 | 0.86 | 0.99 | 1.28 | 1.26 |

Text S2: Surface Profile Characterisation and Heterogeneity

The main article provides a description of the methods used for the various geometric analyses (of aperture and surface profiles), including roughness and spatial correlation. The Python scripts used for these will be made public and can also directly be requested from the authors. The full extended PSD curves obtained for all fractures, for which results are discussed in the paper, are shown in Figure S2 to Figure S6 below.

The full fractures, as discussed in the main text, were split into four subdomains to probe random behaviour and obtain more data points. Table S3 shows some summary results of these subdomains, some of which are used to generate plots in Figure 8.


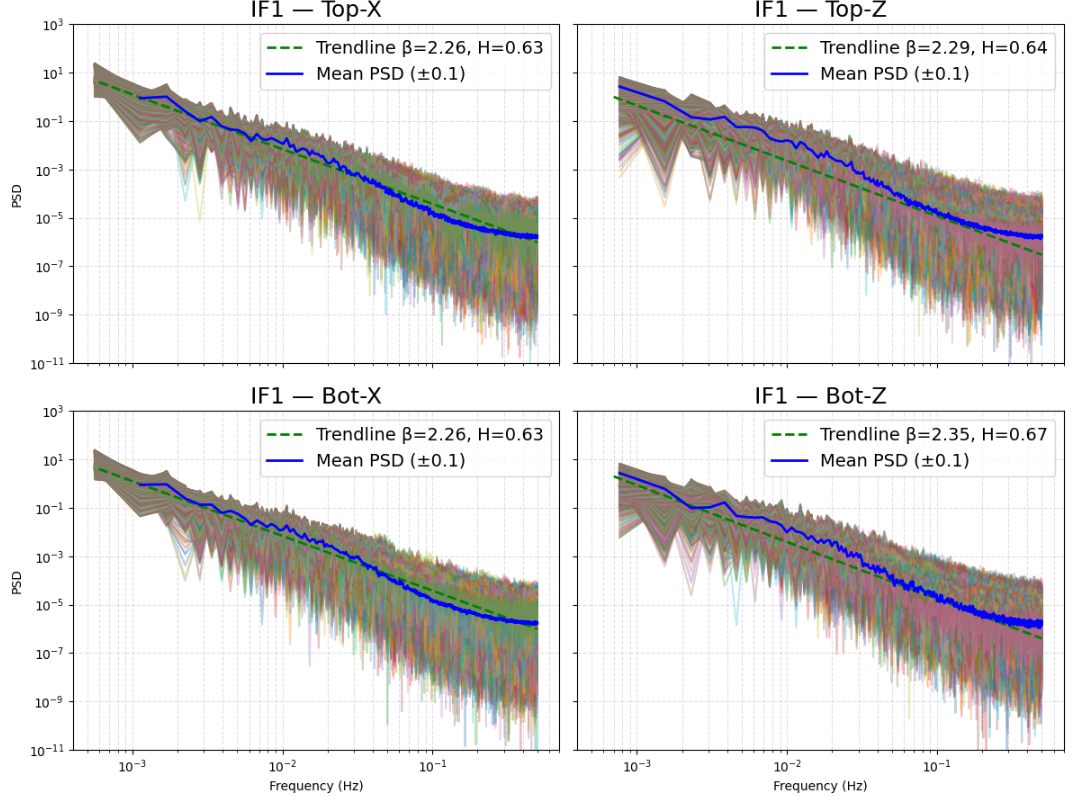


Figure S2: PSD curves for IF1


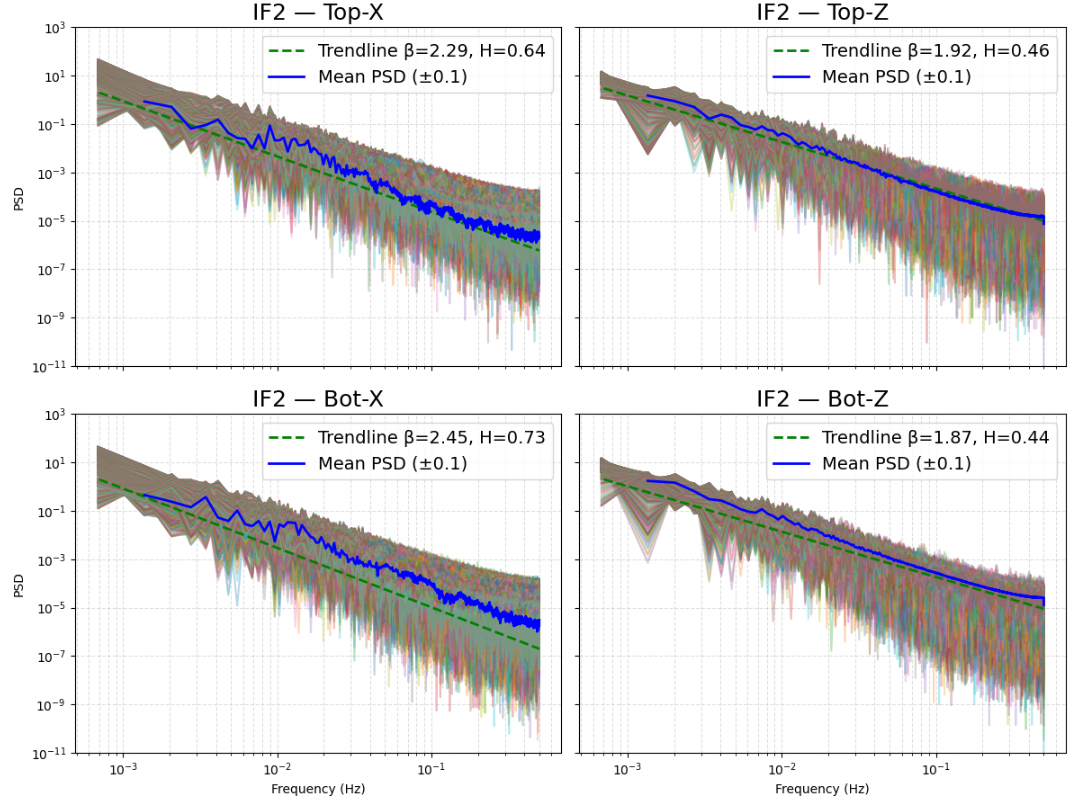


Figure S3: PSD curves for IF2


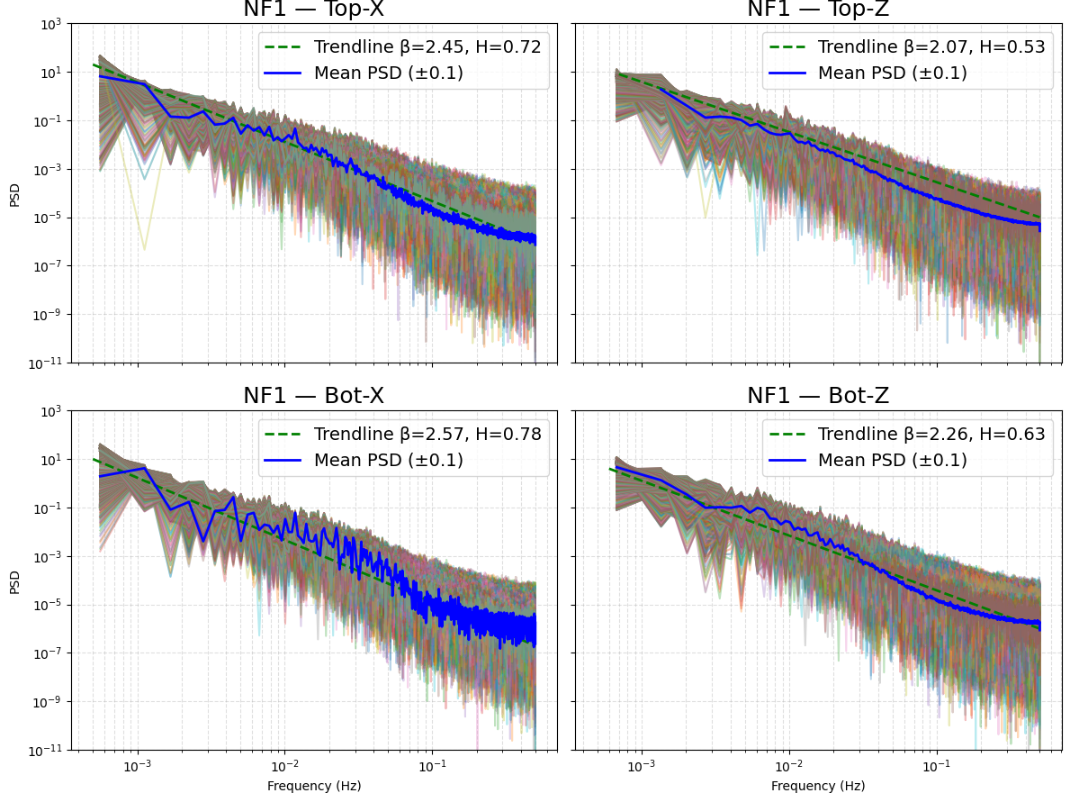


Figure S4: PSD curves for NF1


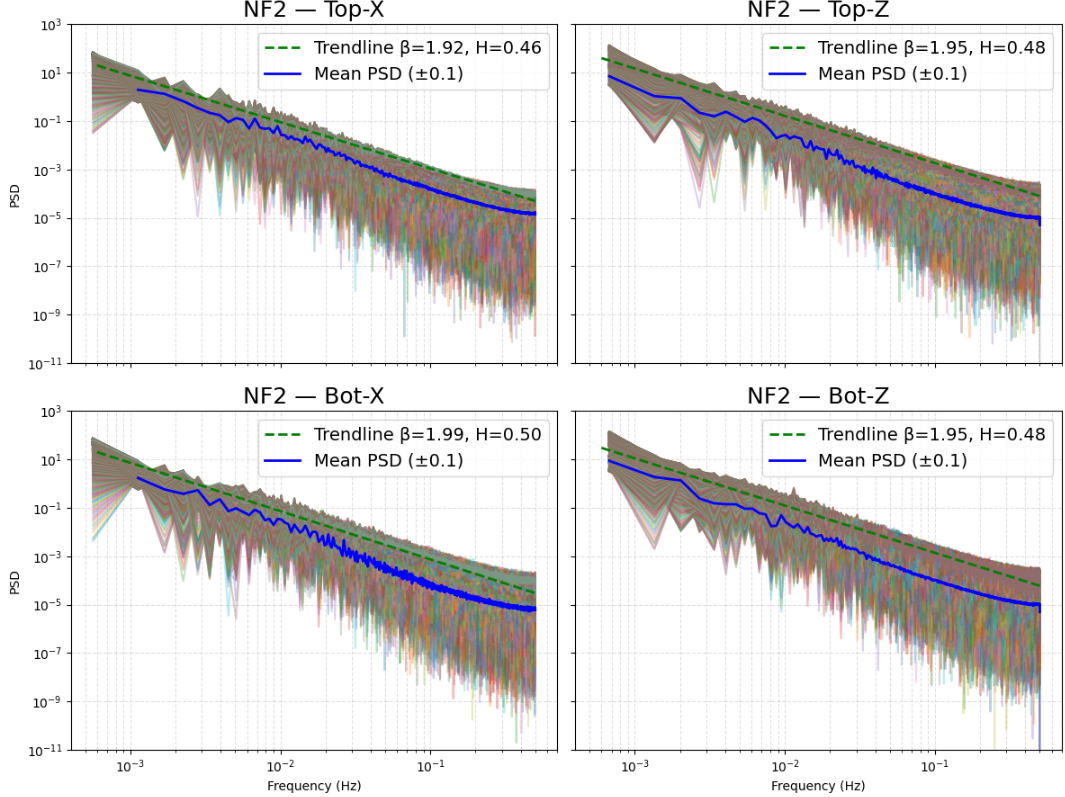


Figure S5: PSD curves for NF2


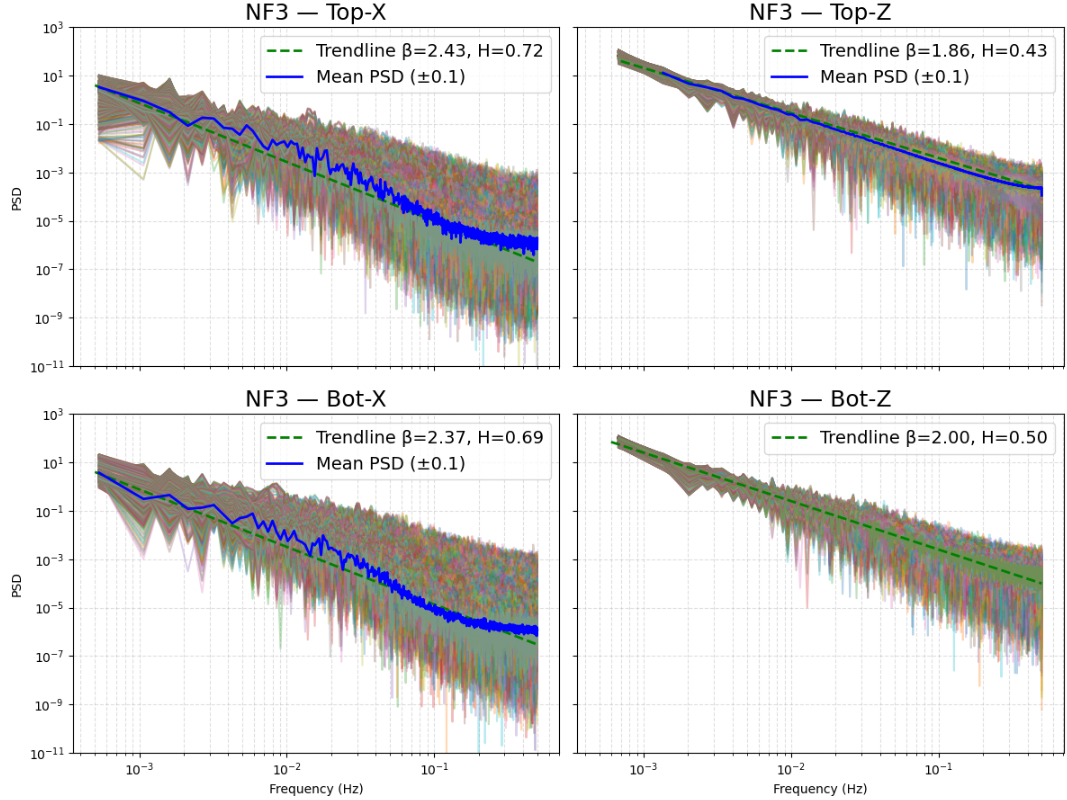


Figure S6: PSD curves for NF3

Table S3: Summary statistics for subdomains of the main fractures. Values from this table are used to generate the plots of correlation length (Lc) against relative roughness (RR) as well as the normalized values using their respective lengths (L). L and Lc are given for both the X and Z directions. The article reports the plot in X-direction, which is perpendicular.

| **Sample**  **ID** | **Subdomain** | **l_x (mm)** | **l_z (mm)** | **Lc_x (mm)** | **Lc_z (mm)** | **lc/l_x** | **lc/l_z** | **RR** |
| --- | --- | --- | --- | --- | --- | --- | --- | --- |
| IF1 | IF1_01 | 2.44 | 1.79 | 0.23 | 0.14 | 0.09 | 0.08 | 0.25 |
| IF1 | IF1_02 | 2.44 | 1.79 | 0.34 | 0.14 | 0.14 | 0.08 | 0.28 |
| IF1 | IF1_03 | 2.44 | 1.79 | 0.15 | 0.15 | 0.06 | 0.08 | 0.25 |
| IF1 | IF1_04 | 2.44 | 1.79 | 0.12 | 0.2 | 0.05 | 0.11 | 0.22 |
| IF2 | IF2_01 | 2 | 2.04 | 0.23 | 0.29 | 0.12 | 0.14 | 0.43 |
| IF2 | IF2_02 | 2 | 2.04 | 0.25 | 0.26 | 0.13 | 0.13 | 0.41 |
| IF2 | IF2_03 | 2 | 2.04 | 0.18 | 0.37 | 0.09 | 0.18 | 0.4 |
| IF2 | IF2_04 | 2 | 2.04 | 0.21 | 0.16 | 0.11 | 0.08 | 0.55 |
| NF1 | NF1_01 | 2.46 | 2.04 | 0.25 | 0.4 | 0.1 | 0.2 | 0.43 |
| NF1 | NF1_02 | 2.46 | 2.04 | 0.4 | 0.32 | 0.16 | 0.16 | 0.6 |
| NF1 | NF1_03 | 2.46 | 2.04 | 0.37 | 0.41 | 0.15 | 0.2 | 0.52 |
| NF1 | NF1_04 | 2.46 | 2.04 | 0.4 | 0.4 | 0.16 | 0.2 | 0.54 |
| NF2 | NF2_01 | 2.44 | 2.04 | 0.89 | 0.45 | 0.36 | 0.22 | 0.36 |
| NF2 | NF2_02 | 2.44 | 2.04 | 0.6 | 0.5 | 0.25 | 0.25 | 0.51 |
| NF2 | NF2_03 | 2.44 | 2.04 | 0.74 | 0.62 | 0.3 | 0.3 | 0.41 |
| NF2 | NF2_04 | 2.44 | 2.04 | 0.72 | 0.89 | 0.3 | 0.44 | 0.42 |
| NF3 | NF3_01 | 2.58 | 2.04 | 0.7 | 0.73 | 0.27 | 0.36 | 0.7 |
| NF3 | NF3_02 | 2.58 | 2.04 | 1.1 | 0.69 | 0.43 | 0.34 | 0.96 |
| NF3 | NF3_03 | 2.58 | 2.04 | 0.8 | 0.9 | 0.31 | 0.44 | 0.67 |
| NF3 | NF3_04 | 2.58 | 2.04 | 0.43 | 0.74 | 0.17 | 0.36 | 0.45 |

Text S3: Mesh Generation

The 3D binary image previously obtained was converted into a surface mesh using iso-surface extraction techniques. This surface mesh was then imported into SnappyHexMesh, an OpenFOAM meshing utility, to create a conformal, body-fitted volumetric mesh that accurately represents the complex topology of the fracture, including its variable aperture field. The mesh cell size was kept at voxel size of 2.75 µm and dimensions as the image (Table S2) to allow for a full resolution. Boundary patches were defined for the inlet, outlet, and walls, with each boundary face characterized by its area $A_{face}$ and outward unit normal vector $n_{f.}$ An example of this mesh is shown on Figure S7.


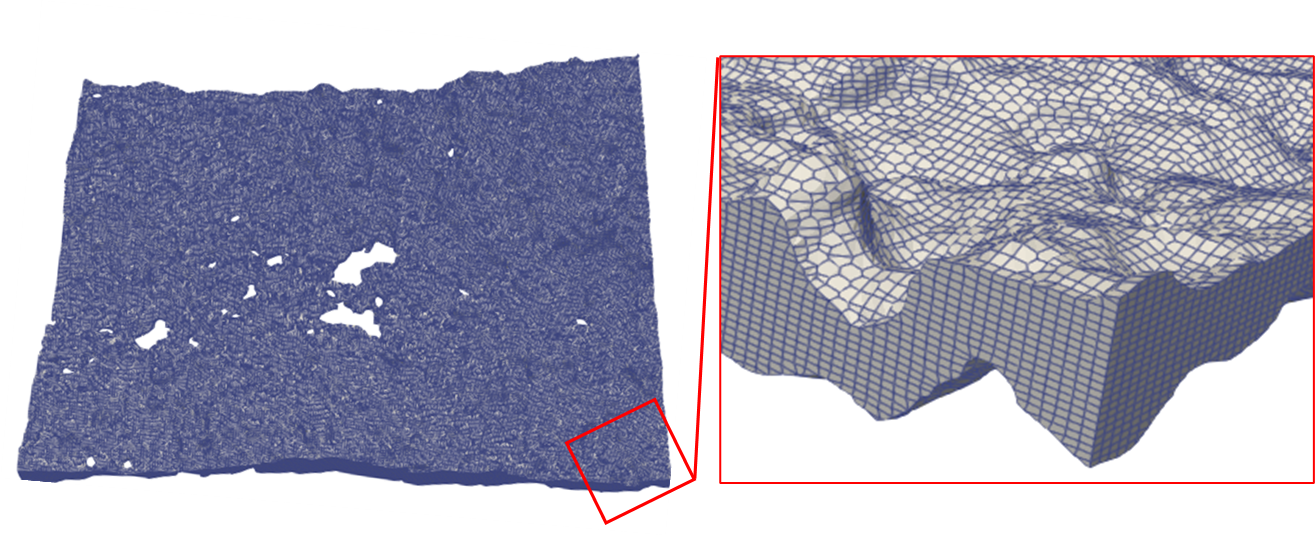


Figure S7: Example of a fracture (NF1) mesh for flow simulation

**Text S5: Numerical Implementation**

The equations were implemented using the simpleFoam solver of GeoChemFoam software (Maes & Menke, 2021), which utilizes a finite volume method with second-order accuracy in space (Gauss linear corrected for the Laplacian term). The pressure-velocity coupling is resolved using the SIMPLE algorithm, ensuring stable convergence of the solution. The governing equations are discretized by integrating over each finite-volume cell and transforming volume integrals into face fluxes via Gauss' theorem. Specifically, the diffusion term $\mu\nabla^{2}u$ was discretized as a sum over faces of the normal velocity gradient multiplied by $A_{face}$. Boundary conditions were applied as follows:

***Inlet:*** A fixed volumetric flow rate 𝑄_set_ (3.3E-12 m^3^/s from experiments) was imposed using the fixedFluxPressure boundary condition. This condition adjusts the inlet pressure to achieve the specified flow rate, ensuring mass conservation.

***Outlet:*** A fixed pressure boundary condition was applied, set to zero-gauge pressure, serving as the reference point for pressure measurements.

***Walls:*** No-slip boundary conditions were enforced, setting the velocity to zero at the solid boundaries, thereby accurately capturing the effects of surface roughness on flow.
